# Automaticity revisited: when print doesn't activate semantics

**DOI:** 10.3389/fpsyg.2015.00117

**Published:** 2015-02-10

**Authors:** Elsa M. Labuschagne, Derek Besner

**Affiliations:** Cognition and Perception Unit, Psychology Department, University of WaterlooWaterloo, ON, Canada

**Keywords:** automaticity, semantic Stroop, spatial attention, visual word recognition, spatial cueing

## Abstract

It is widely accepted that the presentation of a printed word “automatically” triggers processing that ends with full semantic activation. This processing, among other characteristics, is held to occur without intention, and cannot be stopped. The results of the present experiment show that this account is problematic in the context of a variant of the Stroop paradigm. Subjects named the print color of words that were either neutral or semantically related to color. When the letters were all colored, all spatially cued, and the spaces between letters were filled with characters from the top of the keyboard (i.e., 4, #, 5, %, 6, and *), color naming yielded a semantically based Stroop effect and a semantically based negative priming effect. In contrast, the same items yielded neither a semantic Stroop effect nor a negative priming effect when a single target letter was uniquely colored and spatially cued. These findings (a) undermine the widespread view that lexical-semantic activation in word reading is *automatic* in the sense that it occurs without intention and cannot be derailed, and (b) strengthens the case that both implicit and explicit forms of visual word recognition require spatial attention as a necessary preliminary to lexical-semantic processing.

## Introduction

Many cognitive psychologists and cognitive neuroscientists view the automatic-controlled distinction to be of fundamental importance (this distinction can be found in many contemporary cognitive textbooks; e.g., Ashcraft and Klein, 2010; Galotti et al., 2010; Goldstein, 2011; Levitin, 2011; Matlin, 2013). We concern ourselves here with the automatic processing side of this distinction in which such processing is often defined as unconscious, occurring without intention, ballistic (cannot be stopped once started), and needing no capacity or attention of any kind (e.g., Posner and Snyder, 1975; Neely and Kahan, 2001). The only role for attention is to direct the products of such processing (e.g., selection for action). This view is related to late-selection accounts in which all the contents of competing sources are analyzed without attention—up to and including semantics. Attention plays a role *after* such processing (e.g., Deutsch and Deutsch, 1963; Norman, 1969).

Moors and De Houwer (2006) argue (correctly, we believe) that not all of these characteristics need to be simultaneously present for some process to be considered automatic. This conclusion lends weight to the importance of being clear about which particular characteristic of automaticity is being considered. Here we restrict ourselves to the criteria in which an automatic process is triggered without intent, and can neither be stopped nor interfered with by other processes.

### The processing of print

The present work concerns itself with the processing of print in the context of the Stroop task, and the question of whether lexical-semantic processing can be considered automatic in the senses noted above. At present, visual word recognition is widely assumed to be automatic:

… the Stroop effect demonstrates that both the name and *meaning* [italics ours] of a word are processed by skilled readers even when they are trying hard not to process them.

Rayner and Pollatsek (1989, p. 72)

A fail-safe demonstration of automaticity, in particular the automatic nature of accessing word *meaning* [italics ours], involves the Stroop task.

Ashcraft (1994, p. 72)

Indeed, it is fair to say that the assumption of automated word recognition in the mature reader is the “standard” or “received” view in cognitive science, in part because of the impact exerted by results from the Stroop task.

Brown et al. (2002, p. 220)

… these results improve our understanding of the automaticity of *semantic activation* [italics our], as they add to the growing body of evidence suggesting that semantic activation in the Stroop task is indeed automatic and ballistic, in the sense that it occurs without intent and cannot be prevented…

Augustinova and Ferrand (2012, p. 525)

### Spatial attention and visual word recognition

Despite all the strong claims as reflected in the above quotes, the last several decades have seen renewed interest in whether spatial attention plays a role in lexical-semantic processing of print when the task is to explicitly identify a word. Currently, the dominant view is that if spatial attention is not focused on the right location(s) then neither semantic nor lexical processing occurs (McCann et al., 1992; Lachter et al., 2004; Besner et al., 2005; Lien et al., 2010; Waechter et al., 2011). In short, spatial attention is seen as a necessary preliminary to lexical-semantic processing.

This work on the *explicit* processing of print has yet to be integrated into the field's consciousness with respect to the *implicit* processing of print in the context of the Stroop paradigm. For example, Augustinova and Ferrand (2014) consideration of the automaticity of word reading (and semantic activation in particular) in the context of the Stroop paradigm makes no mention of this spatial attention literature, except to note Brown et al. (2002) claim that visual word recognition occurs in the absence of spatial attention.

### Spatial attention and visual word recognition in the context of the Stroop paradigm

The picture is less clear when it comes to the relation between spatial attention and lexical-semantic processing of print in the context of the Stroop paradigm. This literature shows pervasive lexical-semantic effects of a distractor word when the task is to name a color and ignore a distractor color word that is physically separated from the color patch (e.g., Brown et al., 2002; Lachter et al., 2008; Waechter et al., 2011, Experiment 5). Consequently, some authors argue that such distractor processing, despite spatial attention being cued to a different location in space, is strong evidence for word identification without (spatial) attention (Brown et al., 2002; Lachter et al., 2008).

In contrast, Waechter et al. (2011) proposed a different account of these data in which color processing is typically less demanding of spatial attention than is word processing. The consequence is that spatial attention is more distributed and hence the distractor word gets processed—but *with* spatial attention. Evidence for this proposal is provided by Robidoux et al. (2014) who showed that the magnitude of the spatial cueing effect (widely taken as a measure of spatial attention) is significantly smaller for color naming than for reading words aloud. This result is consistent with the hypothesis that visual word recognition makes more demands on spatial attention than does color naming, and hence that spatial attention may be more widely distributed when color naming (even when the spatial cue is 100% valid) than when word reading. Consequently, color naming may afford processing of a distractor word appearing in a different spatial location.

### Distribution of spatial attention *within* a word in the context of the Stroop paradigm

The debate as to whether lexical-semantic processing is automatic or not has also been pursued with spatial attention manipulations *within* a word in the context of the Stroop paradigm. For example, Besner et al. (1997) reported a significant reduction in the size of the Stroop effect when only a single letter in a word was colored compared to when the whole word was colored. Further, Besner and Stolz (1999) reported that spatially pre-cueing a single letter in uniformly colored words produces a significantly smaller Stroop effect than pre-cueing all the letters (Experiments 1 and 2). This finding suggests that word recognition depends on the distribution of spatial attention across the letters in a word. Besner and Stolz (1999; Experiments 3 and 4) also reported that the Stroop effect could be eliminated by coloring the pre-cued letter differently from the un-cued letters and requiring the subject to identify the color of that spatially cued letter. These findings are inconsistent with the widespread view that word recognition is automatic in the sense that lexical and semantic information is inevitably completely activated by presentation of a word.

To be sure, the Besner and Stolz conclusions have been challenged in several quarters. One important issue is whether a reduction in the size of the Stroop effect demands the interpretation that *semantic* processing has been derailed or whether other processes have been compromised instead. In particular, Augustinova and Ferrand (2014) raise a number of methodological and theoretical objections. They conclude that *semantic processing* is automatic whereas response competition associated with lexical processing is not:

… even a complete elimination of Stroop interference does not necessarily guarantee that word reading has been prevented because such a reduction might once again simply reflect the elimination of response conflict rather than the elimination of semantic conflict.

Augustinova and Ferrand (2014, p. 344)

… the methodological and empirical arguments discussed here clearly indicate that no empirical evidence from the Stroop task currently contradicts the widespread automatic view of word reading. Word reading can therefore be conceptualized as a process that can be neither prevented nor controlled.

Augustinova and Ferrand (2014, p. 347)

On the other hand, Manwell et al. (2004) reported an experiment in which cueing and coloring a single target letter in neutral (e.g., “keg”) and color-associated (e.g., “sky”) words eliminated a semantically based Stroop effect. This result has been challenged by Augustinova et al. (2010); they failed to eliminate or even reduce a semantically based Stroop effect with single-letter cueing and coloring. However, if spatial attention is important for word processing (as argued earlier), a potentially critical difference between these experiments is that the stimuli in Augustinova et al. (2010) did not include empty spaces between letters whereas the stimuli in Manwell et al. (2004) did. Indeed, none of the research reviewed by Augustinova et al. (2014) has employed a condition in which there were empty spaces between letters in the word. Such spacing may be critical for preventing letters adjacent to the cued one from being processed (if spatial attention is not narrowly tuned enough) and, given the typically small stimulus set, counteract the activation of enough letters to identify the word^1^.

### The present investigation

#### Semantic stroop

**Figure 1:**
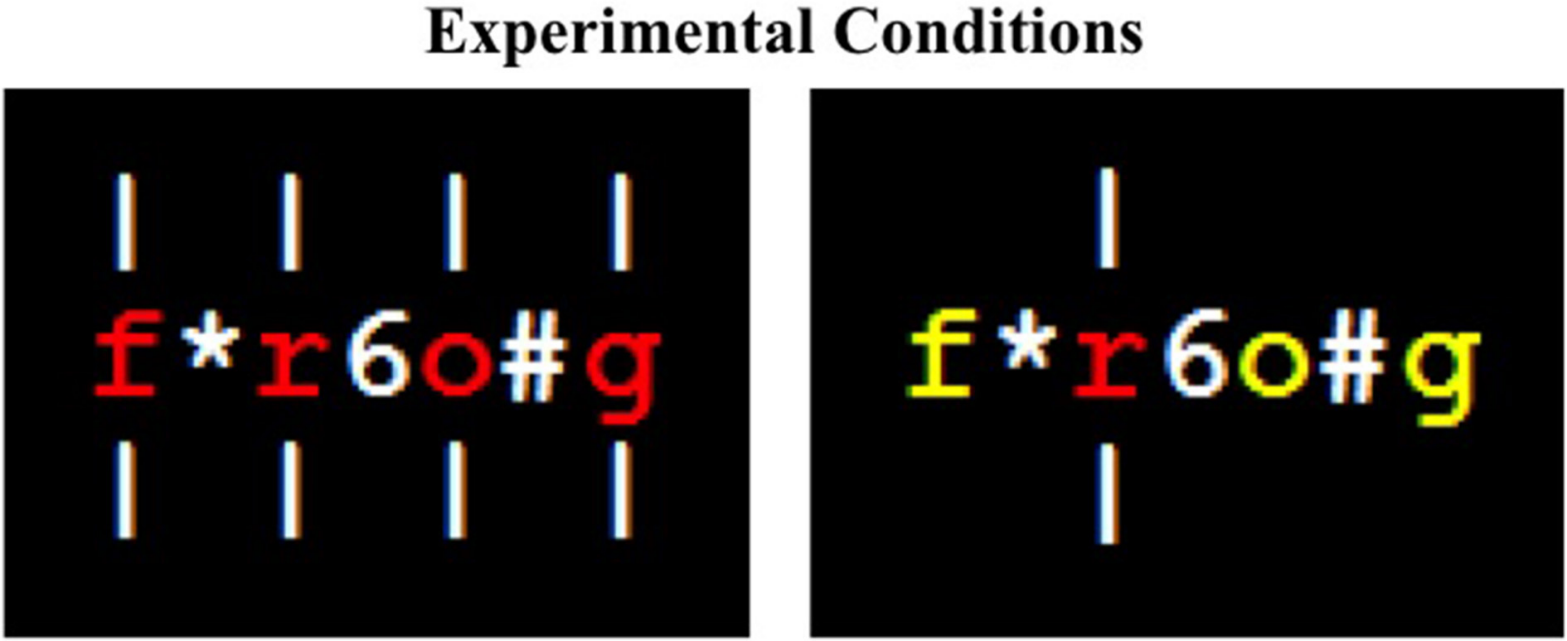
**Sample stimuli from the *all-letters spatially cued and colored* condition (left) and from the *single-letter spatially cued and colored* condition (right)**.

Here we return to the issue of whether semantic processing in the context of the Stroop paradigm can be prevented. We combined three elements in a *semantic* version of the Stroop task which compared incongruent items with neutral ones. In one block of trials, participants named aloud the color in which neutral (e.g., “keg”) and color-associated (e.g., “sky”) words were presented when (a) all letters appeared in one color, (b) all letters were spatially cued, and (c) non-letter characters filled the spaces between adjacent letters. This block constituted the *all-letters cued/colored* (i.e., the homogeneous) condition. In another block of trials, consisting of the same neutral and color-associated words, participants named aloud the color of a single letter when (a) all but one letter appeared in the same color, (b) only the odd-color-out letter was cued, and (c) non-letter characters again filled the spaces between adjacent letters. This block constituted the *single-letter cued/colored* (i.e., the odd-color-out) condition. Examples of these conditions can be seen in Figure 1.

The hypothesis explored here is that combining all these elements serves to focus readers' spatial attention narrowly enough to prevent lexical-semantic processing of the word. Previous experiments have used single letter coloring and spatial cueing (e.g., Besner and Stolz, 1999; Manwell et al., 2004), but all conditions were randomly intermixed in a single block, and, as noted earlier, Augustinova et al. (2010) claim that semantic activation is unaffected by these manipulations. Here, non-letter characters were inserted between letters to maximize the effectiveness of the spatial cue as spatial attention may not, normally, be sufficiently narrow as to focus on only the cued letter^2^. If non-letter characters were inadvertently processed there is no reason to think this would affect semantic processing *per se* in either the neutral or semantically related conditions. Relatedly, the homogenous vs. odd color out conditions were blocked so as to maximize an attentional set in which spatial attention is focused vs. distributed. If the present experiment produces a semantic Stroop effect in the *all-letters cued/colored* condition, but not in the *single-letter cued/colored* condition, then the combination of these two outcomes supports the idea that lexical-semantic processing has been derailed when spatial attention is focused rather than distributed.

#### Negative priming

While the absence of a semantic Stroop effect is consistent with the absence of lexical-semantic processing, caution is indicated because a null effect may not reflect an exhaustive measure of processing. A negative priming effect is observed if responses are slower on trials that are preceded by color-associated words that *match* the font color of the current stimuli. Negative priming therefore serves as another indicator of whether the irrelevant word was processed at the lexical-semantic level. A more detailed description follows in the Results section.

To anticipate the results of the present experiment, there was both a semantically based Stroop effect and a semantically based negative priming effect in the all-letters cued/colored condition. In contrast, neither of these effects was significant in the single-letter cued/colored condition.

## Methods

### Participants

Forty-two students from the University of Waterloo participated in the experiment. Each participant was tested individually and received either course credit or monetary remuneration for participating. All participants had normal or corrected-to-normal vision, as well as normal color vision.

### Stimuli

The stimuli consisted of the neutral words *keg, jail, table*, and *palace*, and the color-associated words *sky, frog, lemon*, and *tomato*, taken from Manwell et al. (2004). These items were matched for length and frequency with the color words in the response set: red, blue, green, and yellow. Items were presented individually, in lowercase, with the spaces between letters filled with a mixture of the characters #, %, and * from the top of the keyboard, and the numbers 4–6. Stimuli were presented in Courier New font, size 18. The characters separating the letters were presented in white (RGB: 255, 255, 255) and the letters were colored using the four colors from the response set: red (RGB: 255, 0, 0), green (RGB: 0, 255, 0), blue (RGB: 0, 40, 255), and yellow (RGB: 255, 255, 0).

In the all-letters cued/colored condition, all letters were presented in the same color, with color-associated trials always presented in an incongruent color (e.g., *sky* presented in green, as opposed to blue). In the single-letter cued/colored condition, a target letter (any letter except the first or last letter of the word) was colored in one color and the remaining letters were all colored in another color from the response set. In this condition, both colors were incongruent with color-associated words. Spatial cueing was also a factor; this is described below.

### Design

The experiment consisted of a 2 cued/colored (all letters vs. single letter) × 2 relatedness (neutral vs. color-associated) (within-subject factors) × 2 counterbalance (order of block type was between subjects). There were a total of 192 experimental trials, with 48 trials per condition.

### Apparatus

Stimuli were displayed on a 22-in. LG Flatron W2242TQ color monitor. Stimulus presentation and data recording were controlled by E-Prime 2.0 experimental software, which was run on an Ultra Vault PC with an Intel® Core™2 Quad CPU @ 2.40 GHz processor. The display had a refresh rate of 60 Hz and a resolution of 1680 × 1050 pixels, while the screen resolution in E-Prime was set to 640 × 480 pixels. Participant responses were collected via an Altec Lansing microphone headset attached to a voice key assembly. Response times were measured to the nearest millisecond.

### Procedure

Participants were seated approximately 70 cm away from a computer monitor, and were instructed to name aloud the color of the target letter(s). They were instructed to respond as quickly and as accurately as possible. Each block began with a set of 16 practice trials that were followed by 96 experimental trials. All words were presented such that the center letter, number, or character was at fixation.

At the beginning of each trial a white fixation marker appeared in the center of the screen on a black background for 500 ms. Next, a spatial cue, consisting of a white, vertical line (i.e., “|”), appeared above and below the position(s) where the target letter(s) would appear. The end of each cue that was closest to the cued letter was 9 mm from the center of the screen. In the all-letters cued/colored condition, each letter in the word was cued by vertical lines. In the single-letter cued/colored condition, only the target letter was cued by vertical lines. The participants' task was to name the color of the cued letter(s).

The stimulus word followed the onset of the spatial by 125 ms. The entire display remained on the screen until the participant made a response. Once a response had been made, the screen remained blank until the researcher had coded the response as correct, incorrect, or spoiled (e.g., cough or microphone failure). This was followed immediately by the fixation marker, which remained on the screen for 500 ms and marked the beginning of the next trial.

## Results

The data of two participants were discarded due to a large number of errors (more than 2.5 standard deviations above the grand mean), resulting in a final sample size of 40, with 20 participants in each counterbalance. Percentage errors were calculated after the removal of spoiled trials (6.3%). Reaction time (RT) analyses were conducted on correct responses only. Correct RTs were subjected to an outlier removal procedure in which RTs more than 2.5 standard deviations above or below the mean RT per subject, per condition, were excluded from all analyses (Van Selst and Jolicoeur, 1994). This resulted in 2.4% of correct responses being discarded. Table 1 shows mean RT, 95% confidence interval (CI), and mean percentage error for each condition. Confidence intervals were computed following the procedures outlined in Masson and Loftus (2003) for within-subject designs.

**Table 1:** **Mean reaction times (RTs in ms), 95% confidence intervals (± CIs), and percentage error (% E) as a function of relatedness and cueing/coloring**.

**Relatedness**	**All letters cued/colored**			**One letter cued/colored**		
	**RT**	**± CI**	**%E**	**RT**	**± CI**	**%E**
Color-associated	630	6	0.8	690	6	0.7
Neutral	608	6	0.9	689	6	0.9
**Difference**	**22**		**−0.1**	**1**		**−0.2**

### Semantic Stroop effect

Mean RTs were analyzed in a 2 × 2 × 2 mixed factorial analysis of variance (ANOVA) with cued/colored (all letters vs. single letter) and relatedness (neutral vs. color-associated) as within-subject factors, and counterbalance (all-letters cued/colored condition completed first vs. single-letter cued/colored condition completed first) as the between-subject factor. The three-way interaction was not significant [*F*_(1, 38)_ < 1]. The critical interaction between cued/colored and relatedness was significant [*F*_(1, 38)_ = 12.55, *MS_e_* = 343.96, *p* < 0.01], indicating that the size of the semantic Stroop effect differed for the all-letters and single-letter cued/colored conditions. Planned comparisons and Bayes factor (BF) analyses (Rouder et al., 2009) confirmed that the 22 ms semantic Stroop effect was significant in the all-letters cued/colored condition [*t*_(39)_ = 4.93, *SE* = 4.37, *p* < 0.001, BF_alternative_ = 1332], whereas it was absent (1 ms) in the single-letter cued/colored condition [*t*_(39)_ = 0.21, *SE* = 3.45, *p* > 0.8, BF_null_ = 5.74]^3^.

### Errors

Neither the three-way interaction nor the critical two-way interaction (cued/colored × relatedness) was significant in a 2 × 2 × 2 mixed ANOVA of the errors [*F*_(1, 38)_ = 2.44, *p* > 0.1 and *F*_(1, 38)_ < 1, respectively].

### Vincentile analysis

**Figure 2:**
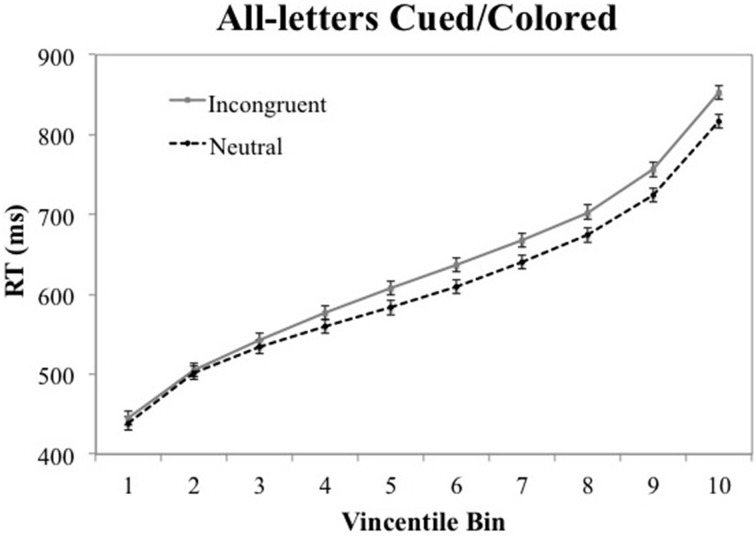
**Vincentile RT means for incongruent (i.e., color-associated) vs. neutral stimuli in the all-letters cued/colored condition with 95% confidence intervals**.

**Figure 3:**
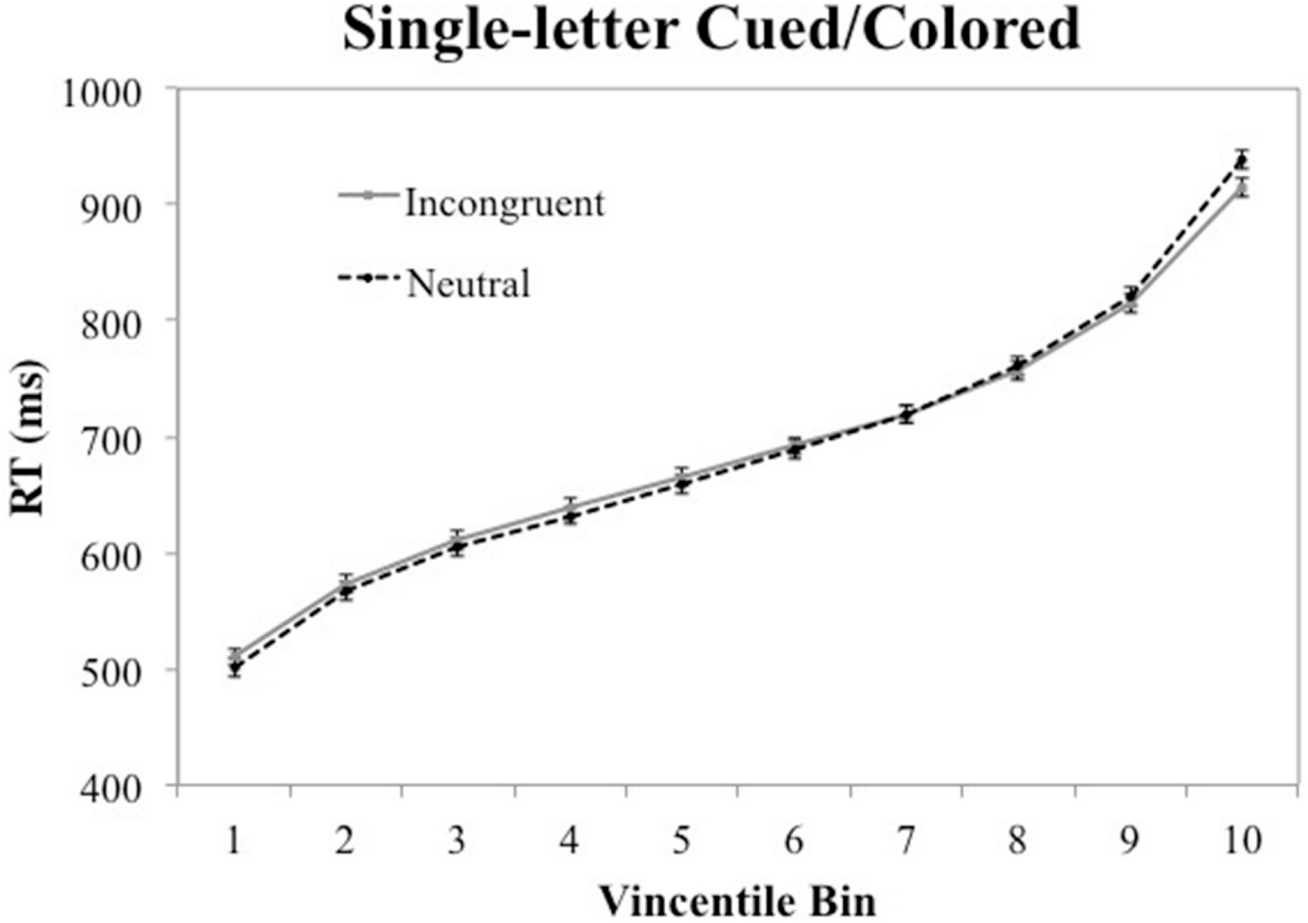
**Vincentile RT means for incongruent (i.e., color-associated) vs. neutral stimuli in the single-letter cued/colored condition with 95% confidence intervals**.

A Vincentizing procedure was used to determine whether the effects seen in the mean RT data, reported above, are seen throughout the RT distributions. First, each participant's RT data were sorted into 10 bins, ranging from their fastest to their slowest responses. The RTs in each percentile range were then averaged across participants to produce mean RTs for the 10 bins in each experimental condition. Figures 2, 3 show the Vincentile plots of RTs and associated 95% CIs for neutral and color-associated (i.e., incongruent) trials for the all-letters cued/colored and single-letter cued/colored conditions, respectively^4^.

Figure 2 illustrates that the semantic Stroop effect in the all-letters cued/colored condition was right-shifted in that it was absent in the first four bins but evident in the last six bins; the interaction of bins × relatedness is *p* < 0.001. Those who believe in automatic semantic processing will need to explain why the effect is right shifted in this specific way since a strong version of this theory would seem to predict this effect to be present throughout the RT distribution. Indeed, Waechter et al, Experiment 5 reported that the standard Stroop effect was seen throughout the RT distribution. (It is interesting that, in a follow up experiment, we see this same pattern (no semantic Stroop effect in the early part of the distribution) in the absence of spatial cueing and non-alphabetic characters between letters).

In contrast, Figure 3 illustrates that the semantic Stroop effect was absent from all 10 bins in the single-letter cued/colored condition. However, the incongruent condition was faster than the neutral condition in the very last bin. This latter result likely reflects a Type I error^5^.

### Negative priming effect

The lack of a semantic Stroop effect in the single-letter cued/colored condition suggests that word processing did not occur. However, as noted earlier, it is important to be cautious when drawing conclusions based on the absence of an effect. Negative priming refers to how the stimulus on the *previous* trial affects the processing of the stimulus on the *current* trial (e.g., Marí-Beffa et al., 2000; Besner, 2001), and is thus another indicator of whether the irrelevant word was processed. A negative priming analysis of the present data is reported below.

All correct trials that were preceded by a color-associated trial to which the response was correct were coded as either *related* or *control*. The stimulus on a *related* trial had a font color that was semantically related to the color-associated word on the previous trial (e.g., a current stimulus presented in blue font preceded by the stimulus word “sky”). In contrast, the stimulus on a *control* trial had a font color that was unrelated to the color-associated word on the previous trial (e.g., a current stimulus presented in green font preceded by the stimulus word “sky”). Both neutral and color-associated trials were included in this analysis to maximize the number of observations.

Table 2 shows the mean RTs and 95% CIs for the negative priming analysis. The RTs that were classified as *related* or *control* were analyzed in a 2 × 2 within-subject ANOVA, with cued/colored (all letters vs. single letter) and trial type (related vs. control) as factors. Critically, there was a significant interaction between cued/colored and trial type [*F*_(1, 39)_ = 7.34, *MS_e_* = 758.34, *p* < 0.01], indicating that the size of the negative priming effect differed significantly for the all-letters and single-letter cued/colored conditions. Indeed, planned comparisons and Bayes factor (BF) analyses (Rouder et al., 2009) revealed a significant 30 ms negative priming effect in the all-letters cued/colored condition [*t*_(39)_ = 4.60, *SE* = 6.63, *p* <.001, BF_alternative_ = 516]. Critically, the 7 ms difference between related and control trials in the single-letter cued/colored condition was not significant [*t*_(39)_ = 1.12, *SE* = 6.15, *p* > 0.25, BF_null_ = 3.28]^6^.

**Table 2:** **Mean reaction times (RTs in ms) and 95% confidence intervals (± CIs) as a function of negative priming condition (related vs. control) and cueing/coloring**.

**Negative priming**	**All letters cued/colored**		**One letter cued/colored**	
**Condition**	**RT**	**± CI**	**RT**	**± CI**
Related	642	9	701	9
Control	612	9	694	9
**Difference**	**30**		**7**	

## Discussion

In summary, a semantically based Stroop effect (22 ms, *CI* = 6) was observed in the present experiment when (a) all the letters in the target word were homogeneously colored, (b) non-letter characters from the top of the keyboard filled the spaces between letters, (c) all letters were spatially cued, and (d) this condition was blocked. In contrast, the semantically based Stroop effect was eliminated (1 ms, *CI* = 6) when only a single target letter in the word was spatially cued and uniquely colored and this condition was blocked. Relatedly, a semantically based negative priming effect was observed when all letters were cued and homogenously colored (30 ms, *CI* = 9 ms), whereas it was absent when only a single letter was cued and uniquely colored (7 ms, *CI* = 9 ms).

**Figure 4:**
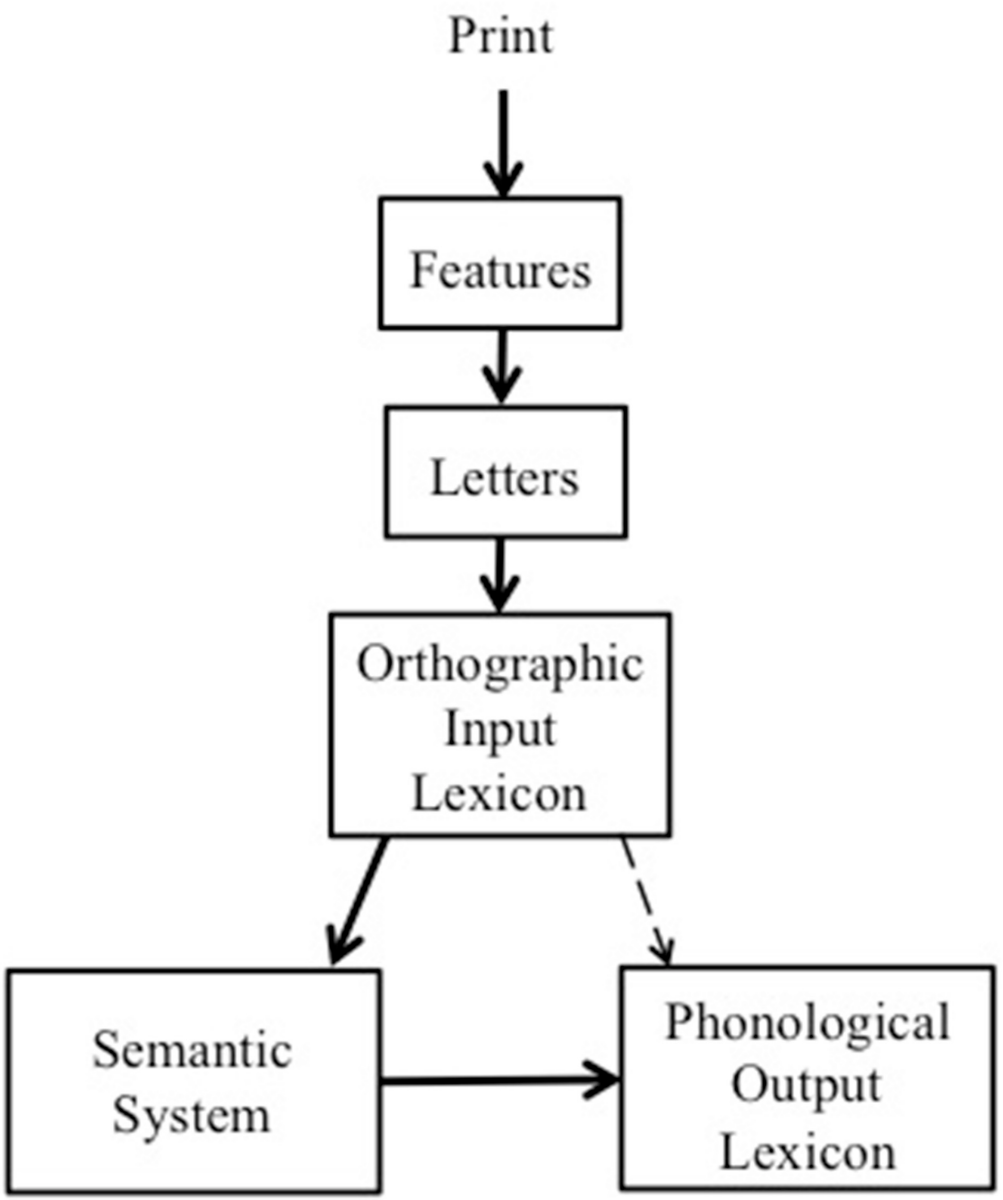
**Five components of the visual word recognition system**. The dashed line indicates the locus of impaired processing due to single letter coloring and spatial cueing as in Augustinova and colleagues' account.

These findings undermine the widely accepted view that lexical-semantic processing is *automatic* in the sense that it occurs without intention and cannot be prevented from occurring. Of course, the present results do not inform us whether semantic activation itself did not occur because it was blocked (i.e., lexical level activation occurred but not semantic activation), or because prior lexical level activation did not occur and hence subsequent semantic activation could not occur. In the latter case there is no challenge to the Neely and Kahan (2001) position, since their claim is that semantic activation is automatic provided that lexical level processing takes place (indeed, Neely and Kahan conceded that spatial attention is a necessary preliminary to lexical processing). In contrast, Augustinova and colleagues' position is undermined by the present findings because their account asserts that single letter coloring and cueing only affects post-lexical processing that does not activate semantics. That is, feature and letter level processing and orthographically based lexical processing is intact, as is the route from that level of representation to semantic activation, but, the *output* from intact orthographic lexical processing is diluted in its ability to activate the phonological lexical level which produces a competing word response. This view is illustrated in Figure 4.

### Future directions

Augustinova et al. (2014) report the results of a Stroop experiment (with both standard and semantic components) in which both RT and an event related potential (ERP) was measured. They found, as before, that single letter coloring and spatial cueing (note however that there were no blank spaces between letters) reduced the magnitude of the standard Stroop effect, but had no impact on the magnitude of the semantic component. Critically, they also concluded that their ERP measure (N_inc_, occurring around 450 ms) reflects semantic conflict rather than response or general conflict. The stage is thus set for an investigation that repeats the essentials of the experiment reported here, and includes the ERP measure used by Augustinova and colleagues. If the both the behavioral data and the ERP measure yield no evidence of semantic processing in the single letter condition then both behavioral and neural indices support the idea that lexical-semantic processing can be prevented, and hence that at least some processes are not automatic. On the other hand, if a dissociation between behavioral and neural data is observed in which the behavioral data show no evidence of lexical-semantic processing in the single letter condition but the neural measure does show such processing, then it will be important for the field at large to take the level of analysis into account when statements are made as to whether a process should be construed as “automatic” or not.

## Conclusions

The results of the present experiment dovetail with results from other approaches that were noted in the introduction (e.g., when a word is to be explicitly identified [as in reading aloud or lexical decision] and spatial attention is directed to an entire word rather than a single letter in a word, and when there is a distractor word in another location and the target location varies across trials, or when there is no distractor item but the target item varies location across trials (e.g., McCann et al., 1992; Lachter et al., 2004; Besner et al., 2005; Lien et al., 2010; Waechter et al., 2011). That is, our perspective on those results, combined with the present ones, is that they falsify the view that both explicit and implicit visual word recognition occurs without the need for spatial attention as a necessary preliminary to lexical-semantic processing.

### Conflict of interest statement

The authors declare that the research was conducted in the absence of any commercial or financial relationships that could be construed as a potential conflict of interest.

## Footnotes

^1^That said, there was little evidence in Besner and Stolz (1999) that an empty space between letters had much effect on the size of the Stroop effect. Note, however, that this contrast is a between-subject comparison (Experiment 1 vs. 2).

^2^We know that this manipulation (inserting non-letter characters between the letters) does not disrupt “normal” reading processes to any large extent because, as we report, a semantically-based Stroop effect was still observed in the homogeneous color condition. Moreover, the size of this effect was essentially the same as that reported by Augustinova et al. (2010), Augustinova and Ferrand (2012) in experiments which did not use inserted non-letter characters (22 ms in the present experiment and 18 ms in the Augustinova et al., experiments). A reviewer wondered whether processing here wasn't better described as “non-normal.” In our thinking if the word recognition processes were “non-normal” in the present experiment because of the interpolated material between letters, then presumably such processes are not “automatic.” But if they are not automatic then they must be controlled. If they are controlled then this is exactly the place where control would consist of *not* treating the irrelevant word as a word, and hence there would be no semantically based Stroop effect in this baseline condition. Given that there is a semantic Stroop effect in this baseline condition it is difficult to accept the premise that “normal” word recognition processes are not in play here.

^3^The absence of a Stroop effect in this condition cannot be explained away as due to the fact that responding was slower in this condition, since slowing responding in various ways does not affect the semantic Stroop effect (e.g., Augustinova and Ferrand, 2012) nor the Stroop effect (which surely includes a semantic component-e.g., Dunbar and MacLeod, 1984).

^4^The 95% CIs for the Vincentile means were calculated using the *MSerror* term associated with the 2× 10 interaction term (relatedness × bin) from two ANOVAs (computed separately for the *all* − letters cued/ colored condition and the *single* − letter cued/colored condition).

^5^If one excludes the last bin, the same pattern as reported in the ANOVA is observed in that the semantic Stroop effect in the all-letters cued/color condition was 20 ms (*p* < 0.001), whereas the 3 ms difference between incongruent and neutral trials in the single-letter cued/colored condition was not significant (*p*> 0.3).

^6^Vincentile analyses were not carried out for the negative priming data because there were too few observations in the *related* condition for reliable estimates.
